# Supplementary material for: A Suggestion of Converting Protein Intrinsic Disorder to Structural Entropy Using Shannon’s Information Theory
Source: Entropy (Basel). 2019 Jun 14;21(6):591. doi: 10.3390/e21060591 (PMC7515080; doi:10.3390/e21060591)
Supplement: Supplementary file 1 [file entropy-21-00591-s001.pdf]

Supplementary Materials

A Suggestion of Converting Protein Intrinsic Disorder to Structural Entropy using Shannon's Information Theory

Hao-Bo Guo [1,*], Yue Ma [2], Gerald A. Taskan [3], Hong Qin [1,4], Xiaohan Yang [2,3,*] and Hong Guo [2,*]

[1] Department of Computer Science and Engineering, SimCenter, University of Tennessee, Chattanooga, TN 37403, USA; hong-qin@utc.edu
[2] Department of Biochemistry and Cellular and Molecular Biology, University of Tennessee, Knoxville, TN 37996, USA; yma27@vols.utk.edu
[3] Biosciences Division, Oak Ridge National Laboratory, Oak Ridge, TN 37831, USA; tuskanga@ornl.gov
[4] Department of Biology, Geology, and Environmental Science, University of Tennessee, Chattanooga, TN 37403, USA
* Correspondence: haobo-guo03@utc.edu (H.-B.G.); yangx@ornl.gov (X.Y.); hguo1@utk.edu (H.G.)

Figure S1. Profile of structural entropy of the residues in the giant human *Titin* protein (C = 34350).
Appendix, on the derivation of the equations that convert the disorder contents to the probabilities of states (with Figures S2 and S3)
Figure S4. The exponential, gamma, and power law fittings to the structural capacities of the human and JCVI-Syn3.0 proteomes
Table S1. Summaries of the exponential, gamma, and power law fittings of the protein structural capacities of the proteomes studied in this paper
List of 25 selenoproteins in human (*H. sapiens*) proteome, whose disorder contents cannot be predicted by PONDR
List of 8 information-rich proteins from DisProt database (v7.0) and their sequences
Table S2. X-ray structures from PDB with resolutions < 1.5 Å, $R = \infty$ (fully disordered) and $C > 20$ in sequences
Table S3. X-ray structures from PDB with resolutions > 3.0 Å, $R = \infty$ (fully disordered) and $C > 20$ in sequences
Figure S5. Distribution of proteins in the CR-space from 500 randomly built protein sequences with capacity randomly chosen in the range [50,800]. ΣH:ΣI ratio is 1.020 from this random set. The vertical dashed line represents the median capacity of 417 from *H. sapiens* proteome, and the horizontal dashed line is at $R = 1$.

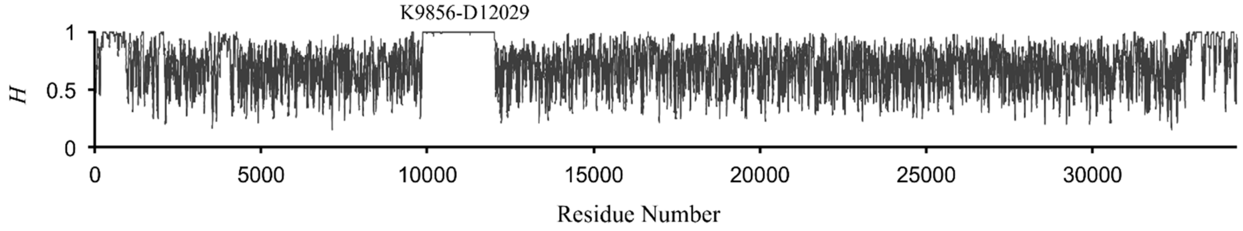

Figure 1. Profile of the structural entropy of the residues in the giant Human *Titin* protein (C = 34350). Residues K9856 to D12029 (2174 AA) are a long intrinsically disordered region (IDR) with $H > 0.95$ for all residues. The composition of residues in this IDR is C: 0, N: 0, A: 129, G: 16, L: 53, I 87, M: 11, V: 331, F: 24, W: 3, S: 35, T: 55, Y: 32, Q: 28, K: 345, R: 64, H: 17, P: 456, D: 13, and E: 475. This region is abundant of disorder-promoting residues including 914 charged residues (K, R, H, D and E) and 456 P.

Appendix

In the present paper the protein intrinsic disorder contents at the residue level are used to quantify the structural entropy and information. The quantities obtained therefore is also limited at the residue level, despite that more sophisticated methods might be able to tackle the structural information at higher (such as atomic and electronic) levels.

The Shannon equation[17] (eq. 1) might be a reasonable choice in studying the structural entropy of a protein since its structure can be viewed as a linear sequence of amino acids linked by peptide bonds. The function H of the Shannon entropy is statistical and derived from the state probabilities (p_i for the i-th state, $i = 1, \ldots, n$, and n is the number of total states) with three original criteria[17] that

1) H is continuous in p_i; i.e., p_i could be any value in range of [0, 1] given that $\sum p_i = 1$;

2) H is a monotonic increasing function when all states are equally distributed with $p_i = 1/n$; it should be noted that H achieves its maximal value of $H_{max} = C = \log n$ in this situation, where C is the capacity;

3) H is additive, which is true for thermodynamic entropies, too. Shannon's definition came from the statistical considerations; i.e., when the choice of a state was split into two states, the original H should be weighted sum of the two individual values of H.

Here, for the structural entropy that concerns the intrinsic order or disorder of proteins, another criterion need be added, i.e.

4) A totally disordered residue contributes the structural entropy of 1, whereas a totally ordered residue contributes zero; the higher the disorder content, the higher the structural entropy a residue has.

Intuitively, criterion 4 fits the definition of both thermodynamic and information entropies. In the former, higher entropy corresponds to higher disorder, and in the latter entropy is synonymous to uncertainty. In both definitions the residues with higher disorder contents should have higher structural entropies. It had been proved[17] that the only H that satisfying criteria 1 to 3 is in the form of eq. 1, and therefore, to use this equation to estimate the structural entropy of a protein the disorder contents of all residues must be converted to probabilities of all states of the protein, in account of the criterion 4.

The disorder predictor gives a vector $\boldsymbol{d} = (d_1, d_2, \ldots d_L)$ that scores the disorder content of each sequence of a protein with L residues. The score d_i of the i-th residue distributes in range of [0,1] with 0 for fully ordered and 1 for fully disordered and that in between for a mixed state. However, considering the structural entropy and information we cannot even treat a single residue as a two-state system (i.e., 0 for the ordered and 1 for the disordered states) and apply eq. 1 such as

$$H(X) = -x\log_2 x - (1-x)\log_2(1-x), \qquad \text{(S1)}$$

where, x is the probability of the first (ordered) state and $(1-x)$ of the second (disordered) state, of that residue. Eq. S1 symmetrically assigns equal contributions to entropy for both states that fits the criterion 2; however, it fails to meet the criterion 4. Instead, the ordered and disordered states should respectively have zero (0) and full (1) contributions, respectively. To fit the criterion 4, we may suppose an imaginary two-state system as shown in Fig. S3A. The two states termed α ($x = 0$) and β ($x = 1$) contribute equally to the structural entropy and the entropy $H(x)$ is zero at both extrema. The fully mixed state at $x = 0.5$ has the maximal entropy of $H(x) = 1$, and this state should be regarded as the disordered state. Similarly, a three-state (or higher dimension) system may be supposed (Fig. S3B) with probabilities x_A for the α-, x_B for the β- and x_C for the c-states, respectively, with $\sum_{i=A,B,C} x_i = 1$. The fully mixed state ($x_i = 1/3$) has the maximal entropy of $H = \log_2 3$.

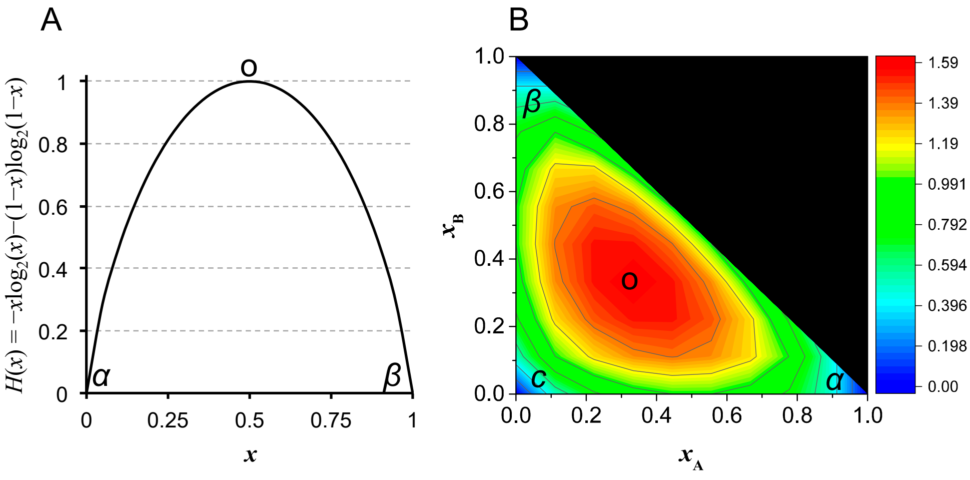

Figure 2. Profiles of Shannon function for (**A**) a two-state system; both α- (x = 0) and β-states (x = 1) have zero entropies whereas the state with maximal entropy of 1.0 at x = (1-x) = 0.5; (**B**) a three-state system. The 2D contour map is a projection onto the probability space of x_A and x_B; the black region is inaccessible with total probabilities larger than 1. All extreme states have zero entropies and the mixed state at $x_A = x_B = x_C$ = 1/3 has the maximal entropy of $\log_2 3$ = 1.585.

Therefore, the criterion 4 shown above gives two alternative approaches for converting the disorder contents ***d*** to probabilities of states. In the first approach, ***d*** is directly used in the estimations, i.e.,

$$H(x_i) = d_i, \qquad \text{(S2)}$$

d_i is the disorder content of the i-th residue. This approach (direct approach) is equivalent to a two-state approach and d_i automatically takes the value between 0 and 1, with 0 for the fully ordered and 1 for the fully disordered, fit well with criterion 4. However, a careful consideration of criterion 2 need be taken because the two extreme states (0 and 1) contribute unevenly to the entropy. Nevertheless, for a protein with L residues the maximal entropy or the capacity of the protein is $H_{max} = L$, when all residues are in the fully disordered state, which is consistent with the total state number of 2^L for the two-state system.

The second approach is based on Shannon's equation (Shannon-approach). Considering the two-state system in Fig. S3A, the α- and β-states (the 0 and 1 states) could be regarded as two representative secondary structures. All mixed states between 0 and 1, therefore, have mixed secondary structural characteristics with the fully mixed state (x = 0.5) having the maximal entropy of $\log_2 2 = 1$. The symmetry of Shannon's function (eq. S1) provides that both states contribute equally to the entropy, and therefore criterion 2 holds. In this approach, the disorder contents are converted to the probabilities of states using

$$H(X) = \sum_{i=1}^{L} -x_i \log_2 x_i - (1 - x_i)\log_2(1 - x_i),$$

$$x_i = d_i/2. \text{ (S3)}$$

In both approaches the capacity C, or the maximal entropy H_{max}, of the protein equals to the residue number L; i.e., the total number of the states of the protein is $n = 2^L$. The difference between the two approaches is that the direct-approach gives a linear function of the disorder content (orange in Fig. S3) and the Shannon-approach is a half function of the Shannon's equation in Eq. S1 (green in Fig. S3). It should be noted from that the disorder contents might underestimate the structural entropies.

The Shannon-approach is adopted in the main text. It should be noted from Fig. S3 that an alternative approach could be derived from the secondary structure predictions either use a two-state or three-state system or in higher dimensions. Moreover, this approach could be assisted by molecular dynamics (MD) simulations by providing an ensemble of configurations from which the probabilities of states could be extracted, which should be promising because the protein dynamics is involved.

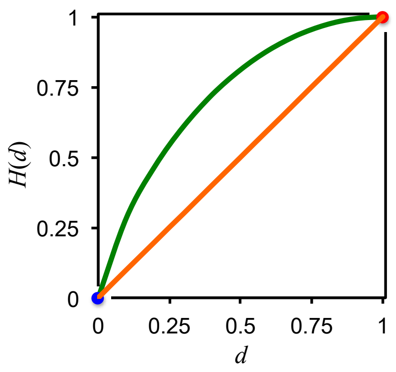

Figure 3. The structural entropy $H(d)$ in function of the intrinsic disorder d. The orange line is from the direct-approach and the green line is from the Shannon-approach. Blue dot stands for the fully ordered state and red dot for the fully disordered state. Both profiles are based on two-state systems. In the direct-approach the two extreme states do not contribute equally to the entropy with the ordered state has entropy of 0 and disordered state has entropy of 1, respectively. In the Shannon-approach the fully ordered state could be served as either of two extreme states with entropies of 0, whereas the fully disordered state with entropy of 1 is the equally mixed state of both extreme states.

The exponential model with $L = Ae^{bx}$, gamma model with $L = \Gamma^{-1}(x/(n+1);\alpha,\beta)$, and power law model with $L = Ax^b$ have been used to fit the protein length L in the proteomes. Here x is the serial number of the protein in the hierarchical rank and n is the total number of proteins in the proteome. A and b are the frequency factors and exponential indexes in the exponential and power law models. The inverse gamma function was applied in the gamma model and the parameters α and β are calculated via

$$\alpha = \left(\sum_{i=1}^{n} L_i\right)^2 / \left[n\sum_{i=1}^{n} L_i^2 - \left(\sum_{i=1}^{n} L_i\right)^2\right],$$
$$\beta = n\sum_{i=1}^{n} L_i^2 / (n-1)\sum_{i=1}^{n} L_i. \qquad \text{(S4)}$$

The coefficient of determination, R^2, was calculated using the standard procedure of

$$R^2 = 1 - \sum_{i=1}^{n} e_i^2 / \sum_{i=1}^{n} (L_i - \bar{L})^2, \qquad \text{(S5)}$$

where, $e_i = f_i - L_i$ is the error for the ith protein, and $\bar{L}$ is the average protein length of the proteome.

Figure S4 shows examples from the human (*H. sapiens*) and bacterial (*JCVI-Syn3.0*) proteomes. The fitting results of all proteomes assessed in the present work are summarized in Table S1. In all cases, the exponential model yield fittings with coefficient R^2 larger than 0.9; the gamma model gives good fittings except for the two animal models surveyed here. The power law model did fit well at the short-L side but had relatively large deviations at the long-L side. We may therefore use the exponential model for the fitting of all proteomes.

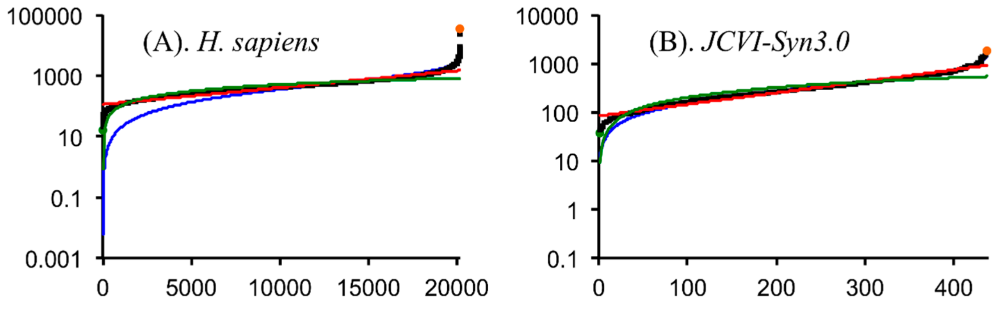

Figure 4. Distribution of protein length L from (**A**) *H. sapiens* (human) and (**B**) *JCVI-Syn3.0* proteomes ranked in a hierarchical order (black dots) fitted with exponential (red), gamma (blue) and power law (green) models. The horizonal axis is the serious number of the proteins hierarchically ranked by the structural capacity, and the vertical length represents the structural capacity of the proteins. The proteins with largest and smallest structural capacities are shown in orange and green dot, respectively.

Table 1. Fitting of the structural capacity *L* using different models.

Species	Exponential[a]			Power law[a]			Gamma		
	A	*b*	R^2	*A*	*b*	R^2	α	β	R^2
H. sapiens	113.7	1.3E-4	0.939	0.844	0.695	0.814	0.858	654.2	0.792
D. melanogaster	94.8	2.0E-4	0.946	0.628	0.752	0.826	0.768	699.9	0.804
S. cerevisiae	102.9	4.4E-4	0.934	1.347	0.733	0.888	1.664	296.9	0.983
A. thaliana	88.0	9.0E-5	0.933	0.419	0.718	0.893	1.779	227.8	0.968
O. sativa	70.5	6.0E-5	0.969	0.206	0.735	0.837	1.418	265.3	0.986
A. trichopoda	59.8	1.0E-4	0.980	0.497	0.668	0.723	1.153	275.0	0.971
P. patens	46.1	1.0E-4	0.986	0.092	0.835	0.788	1.005	350.3	0.977
Lokiarchaeum	55.7	4.8E-4	0.959	0.929	0.710	0.854	1.517	177.0	0.939
I. hospitalis	80.0	1.5E-3	0.961	6.251	0.575	0.834	2.329	119.5	0.981
N. equitans	77.5	4.0E-3	0.961	10.231	0.586	0.811	1.895	147.8	0.940
JCVI-Syn3.0	84.2	5.5E-3	0.961	9.273	0.669	0.850	1.828	194.8	0.982
Rickettsiale	72.9	1.3E-3	0.966	3.987	0.630	0.809	1.681	179.6	0.969
S. elongatus	79.8	9.0E-4	0.957	3.445	0.622	0.857	2.184	139.8	0.991
Mimivirus	81.4	2.5E-3	0.933	4.753	0.690	0.865	1.536	232.3	0.946
Pandoravirus	39.1	1.2E-3	0.990	0.792	0.793	0.793	1.271	203.9	0.980

[a] The functions used for the three models are shown above. For both the exponential and power law models *A* is the frequency factor (or pre-exponential factor) and *b* is the exponential index.

List of 25 selenoproteins in human (H. sapiens) proteome, whose disorder contents cannot be predicted by PONDR

sp|Q99611|SPS2_HUMAN
sp|Q9BQE4|SELS_HUMAN
sp|P49908|SEPP1_HUMAN
sp|P59797|SELV_HUMAN
sp|Q8IZQ5|SELH_HUMAN
sp|Q9Y6D0|SELK_HUMAN
sp|P63302|SELW_HUMAN
sp|O60613|SEP15_HUMAN
sp|Q9BVL4|SELO_HUMAN
sp|Q9NZV5|SELN_HUMAN
sp|P62341|SELT_HUMAN
sp|Q8WWX9|SELM_HUMAN
sp|P02729|GLUR_HUMAN
sp|Q92813|IOD2_HUMAN
sp|P55073|IOD3_HUMAN
sp|P18283|GPX2_HUMAN
sp|P07203|GPX1_HUMAN
sp|P59796|GPX6_HUMAN
sp|P22352|GPX3_HUMAN
sp|P49895|IOD1_HUMAN
sp|Q16881|TRXR1_HUMAN
sp|Q86VQ6|TRXR3_HUMAN
sp|Q9NNW7|TRXR2_HUMAN
sp|Q9C0D9|EPT1_HUMAN
sp|Q9NZV6|MSRB1_HUMAN

List of 8 information-rich (R < 1) proteins from DisProt database (v7.0) and their sequences

Gene	Capacity	Entropy	Info	logC	R
DP00851	256.000	84.528	171.472	8.000	0.493
DP00088	663.000	231.965	431.035	9.373	0.538

DP00925	277.000	109.069	167.931	8.114	0.649
DP00271	348.000	142.439	205.561	8.443	0.693
DP00927	274.000	122.846	151.154	8.098	0.813
DP00974	398.000	188.842	209.158	8.637	0.903
DP00801	52.000	24.706	27.294	5.700	0.905
DP00509	86.000	42.265	43.735	6.426	0.966

>DP00851
MSVTTETTAGAAAGSDAIVDLRGMWVGVAGLNIFYLIVRIYEQIYGWRAGLDSFAPEFQTYWLSILWTEIPLE
LVSGLALAGWLWKTRDRNVDAVAPREELRRHVVLVEWLVVYAVAIYWGASFFTEQDGTWHMTVIRDTDF
TPSHIIEFYMSYPIYSIMAVGAFFYAKTRIPYFAHGFSLAFLIVAIGPFMIIPNVGLNEWGHTFWFMEELFVAPL
HWGFVFFGWMALGVFGVVLQILMGVKRLIGKDCVAALVG
>DP00088
MFGKLSLDAVPFHEPIVMVTIAGIILGGLALVGLITYFGKWTYLWKEWLTSVDHKRLGIMYIIVAIVMLLRGF
ADAIMMRSQQALASAGEAGFLPPHHYDQIFTAHGVIMIFFVAMPFVIGLMNLVVPLQIGARDVAFPFLNNLS
FWFTVVGVILVNVSLGVGEFAQTGWLAYPPLSGIEYSPGVGVDYWIWSLQLSGIGTTLTGINFFVTILKMRAP
GMTMFKMPVFTWASLCANVLIIASFPILTVTVALLTLDRYLGTHFFTNDMGGNMMMYINLIWAWGHPEVYI
LILPVFGVFSEIAATFSRKRLFGYTSLVWATVCITVLSFIVWLHHFFTMGAGANVNAFFGITTMIIAIPTGVKIFN
WLFTMYQGRIVFHSAMLWTIGFIVTFSVGGMTGVLLAVPGADFVLHNSLFLIAHFHNVIIGGVVFGCFAGMT
YWWPKAFGFKLNETWGKRAFWFWIIGFFVAFMPLYALGFMGMTRRLSQQIDPQFHTMLMIAASGAVLIAL
GILCLVIQMYVSIRDRDQNRDLTGDPWGGRTLEWATSSPPPFYNFAVVPHVHERDAFWEMKEKGEAYKKP
DHYEEIHMPKNSGAGIVIAAFSTIFGFAMIWHIWWLAIVGFAGMIITWIVKSFDEDVDYYVPVAEIEKLENQH
FDEITKAGLKNGN
>DP00925
MQKQSLLIHFSKKIVSHRYFTRIIITLILFNALLVGLETYPALRHEYGSLFHVLDVILLWIFTLEILTRFLATTPKK
DFFKGGWNWFDTIIVLSSHIFVGGHFITVLRILRVLRVLRAISVIPSLRRLVDALMLTIPALGNILILMSIIFYIFAV
LGTMLFANVAPEYFANLQLSMLTLFQIVTLDSWGSGVMRPILVDIPWAWTYFIAFVLVGTFIIFNLFIGVIVNN
VEKANEDEVKDKVKEKEEAAQKQMDSLHEELKEIKQYLKSIEKQNRSS
>DP00271
MNGTEGPNFYVPFSNKTGVVRSPFEAPQYYLAEPWQFSMLAAYMFLLIMLGFPINFLTLYVTVQHKKLRTPL
NYILLNLAVADLFMVFGGFTTTLYTSLHGYFVFGPTGCNLEGFFATLGGEIALWSLVVLAIERYVVVCKPMSN
FRFGENHAIMGVAFTWVMALACAAPPLVGWSRYIPEGMQCSCGIDYYTPHEETNNESFVIYMFVVHFIIPLIV
IFFCYGQLVFTVKEAAAQQQESATTQKAEKEVTRMVIIMVIAFLICWLPYAGVAFYIFTHQGSDFGPIFMTIPA
FFAKTSAVYNPVIYIMMNKQFRNCMVTTLCCGKNPLGDDEASTTVSKTETSQVAPA
>DP00927
MSRKIRDLIESKRFQNVITAIIVLNGAVLGLLTDTTLSASSQNLLERVDQLCLTIFIVEISLKIYAYGVRGFFRSG
WNLFDFVIVAIALMPAQGSLSVLRTFRIFRVMRLVSVIPTMRRVVQGMLLALPGVGSVAALLTVVFYIAAVM
ATNLYGATFPEWFGDLSKSLYTLFQVMTLESWSMGIVRPVMNVHPNAWVFFIPFIMLTTFTVLNLFIGIIVDA
MAITKEQEEEAKTGHHQEPISQTLLHLGDRLDRIEKQLAQNNELLQRQQPQKK
>DP00974
MDSSAGPGNISDCSDPLAPASCSPAPGSWLNLSHVDGNQSDPCGPNRTGLGGSHSLCPQTGSPSMVTAITIM
ALYSIVCVVGLFGNFLVMYVIVRYTKMKTATNIYIFNLALADALATSTLPFQSVNYLMGTWPFGNILCKIVISI
DYYNMFTSIFTLCTMSVDRYIAVCHPVKALDFRTPRNAKIVNVCNWILSSAIGLPVMFMATTKYRQGSIDCTL
TFSHPTWYWENLLKICVFIFAFIMPVLIITVCYGLMILRLKSVRMLSGSKEKDRNLRRITRMVLVVVAVFIVCW
TPIHIYVIIKALITIPETTFQTVSWHFCIALGYTNSCLNPVLYAFLDENFKRCFREFCIPTSSTIEQQNSARIRQNT
REHPSTANTVDRTNHQLENLEAETAPLP
>DP00801
MDKVQYLTRSAIRRASTIEMPQQARQNLQNLFINFCLILICLLLICIIVMLL
>DP00509
MIPAVVLLLLLLVEQAAALGEPQLCYILDAILFLYGIVLTLLYCRLKIQVRKAAITSYEKSDGVYTGLSTRNQET
YETLKHEKPPQ

Table 2. X-ray structures from PDB with resolutions < 1.5 Å, R = ∞ (fully disordered) and C > 20 in sequences [a].

PDB ID:chain ID	Resolution (Å)	Description	C	Oligomeric state
1JCD:A	1.3	Ala-zipper	52	Homo-trimer
1K6F:A	1.3	Collagen triple helix	30	Homo-trimer
1RJU:V	1.44	Yeast copper tionein	36	Monomer
1X1K:A	1.1	Host-guest peptide	27	Homo-trimer
2V8F:C	1.1	Profilin-actin complex	21	Monomer
3B0S:A	1.45	Collagen model	27	Homo-trimer
3IPN:A	1.21	Modified collagen	21	Homo-trimer
3WN8:A	1.45	Collagen model	24	Homo-trimer
4GYX:A	1.49	Type-III collage	31	Homo-trimer
4OY5:A	0.89	Collagen model	30	Homo-trimer

a. For identical entries only a unique sequence was chosen for analysis in present work.

Table 3. X-ray structures from PDB with resolutions ≥ 3.0 Å, R = ∞ (fully disordered) and C > 20 in sequences [a].

PDB ID:chain ID	Resolution (Å)	Description	*C*	Oligomeric state
2F6A:E	3.29	Collagen complex	30	Homer-trimer
2V53:B[b]	3.2	SPARC-collagen complex	33	Homo-trimer
3U85:B[b]	3.0	Human menin in complex with MLL1	36	Monomer
4AUO:C	3.0	MMP-1 in complex with collagen	40	Homo-trimer
4BJ3:C	3.042	Integrin alpha2 I-collagen complex	21	Homo-trimer
4BKL:E	3.25	Triple-helical J1 peptide	37	Homo-trimer
4FQ3:B[b]	3.0	Transportin/FUS-NLS	37	Monomer
4GU0:E[b]	3.103	LSD2 with H3	26	Monomer
4GWQ:H	4.5	RNA Pol-II subunit	35	Monomer
4HTV:B	3.0	BFDV Cap NLS peptide complex	29	Monomer
5JXT:Q	3.009	MtISWI bound with histone H4 tail	21	Monomer
5MUB:E	3.1	ACC1 Fab in complex with CG05	33	Monomer
6F5P:G	4.14	Influenza virus transcriptase unit	28	Monomer

a. For identical entries only a unique sequence was chosen for analysis in present work. b. Entry collected in the IDEAL[36] database.

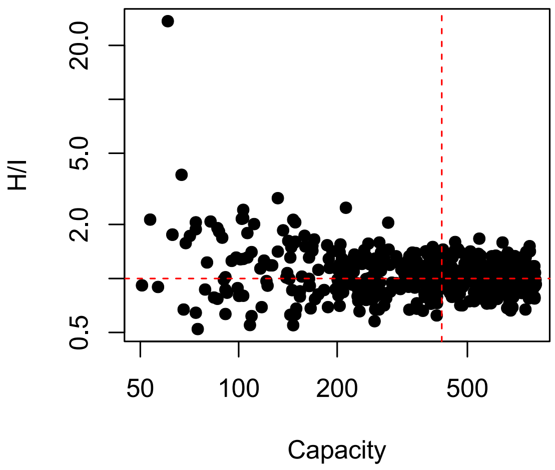

Figure 5. Distribution of proteins in the CR-space from 500 randomly built protein sequences with capacity randomly chosen in the range [50, 800]. *ΣH:ΣI* ratio is 1.020 from this random set. The vertical dashed line represents the median capacity of 417 from *H. sapiens* proteome and the horizontal dashed line is at *R* = 1.
